# M-Cadherin Is a PAX3 Target During Myotome Patterning

**DOI:** 10.3389/fcell.2021.652652

**Published:** 2021-04-01

**Authors:** Joana Esteves de Lima, Reem Bou Akar, Myriam Mansour, Didier Rocancourt, Margaret Buckingham, Frédéric Relaix

**Affiliations:** ^1^Univ Paris Est Creteil, Institut National de la Santé et de la Recherche Médicale (INSERM), EnvA, Etablissement Français du Sang (EFS), Assistance Publique Hopitaux de Paris (AP-HP), Institut Mondor de Recherche Biomedicale (IMRB), Creteil, France; ^2^Department of Developmental and Stem Cell Biology, Institut Pasteur, Paris, France

**Keywords:** PAX3, M-Cadherin, CDH15, myotome, myogenesis

## Abstract

PAX3 belongs to the paired-homeobox family of transcription factors and plays a key role as an upstream regulator of muscle progenitor cells during embryonic development. *Pax3*-mutant embryos display impaired somite development, yet the consequences for myotome formation have not been characterized. The early myotome is formed by PAX3-expressing myogenic cells that delaminate from the dermomyotomal lips and migrate between the dermomyotome and sclerotome where they terminally differentiate. Here we show that in *Pax3*-mutant embryos, myotome formation is impaired, displays a defective basal lamina and the regionalization of the structural protein Desmin is lost. In addition, this phenotype is more severe in embryos combining *Pax3*-null and *Pax3* dominant-negative alleles. We identify the adhesion molecule M-Cadherin as a PAX3 target gene, the expression of which is modulated in the myotome according to *Pax3* gain- and loss-of-function alleles analyzed. Taken together, we identify M-Cadherin as a PAX3-target linked to the formation of the myotome.

## Introduction

Skeletal muscles of the trunk and limbs derive from the somites. The specification of these muscles relies on the paired-homeobox transcription factors PAX3 and PAX7 that are master regulators of myogenesis (Relaix and Marcelle, 2009). PAX3 is expressed in the paraxial mesoderm and after segmentation its expression is maintained in the epithelial somite before being delimited to the dermomyotome (Buckingham and Rigby, 2014). PAX7 expression is initiated later and is restricted to the central dermomyotome, where it is co-expressed with PAX3 to provide the future progenitor cell population of the trunk and limb muscles (Gros et al., 2005; Kassar-Duchossoy, 2005; Relaix et al., 2005). *Pax3*-mutant embryos present a severe muscle phenotype in the trunk and limbs, including reduced epaxial and hypaxial dermomyotome domains, leading to a severely impaired development of the trunk muscles and the complete absence of muscles of migratory origin (Bober et al., 1994; Goulding et al., 1994; Relaix et al., 2003). In contrast, *Pax7*-mutant embryos do not display myogenic defects during development (Seale et al., 2000; Relaix et al., 2004, 2006). This is consistent with the delayed PAX7 expression within PAX3-expressing cells in the mouse embryo, despite partially redundant activities between these two transcription factors (Relaix et al., 2004; Soleimani et al., 2012). The requirement of PAX3 for the proper development of the epaxial and hypaxial dermomyotome domains is associated with its role in ensuring cell survival. In the absence of PAX3, dermomyotomal cells undergo apoptosis (Borycki et al., 1999; Mansouri et al., 2001; Relaix et al., 2004; Buckingham et al., 2006; Zhou et al., 2008). The proper patterning of the somites and myotome relies on several signaling pathways such as FGF, WNT, and SHH that have been identified in complementary studies performed in mouse, chicken and zebrafish embryos (Groves et al., 2005; McDermott et al., 2005; Hamade et al., 2006; Brunelli et al., 2007).

The myotome develops from the PAX3-expressing cells that migrate from the lips of the dermomyotome and intercalate between the dermomyotome and the sclerotome. The early myotome is composed of mononucleated myocytes that act as a scaffold to support further myogenic development contributing to the formation of the muscles of the ribs and the back and of the abdominal muscles (Cinnamon et al., 1999). Skeletal muscle commitment relies on the activation of the myogenic regulatory factors (MRFs), a family of basic helix-loop-helix (bHLH) transcription factors that comprises MYF5, MRF4, MYOD, and MYOG. Cells located at the edges of the dermomyotome activate the expression of the MRFs, first at the epaxial level, where muscle progenitors activate *Myf5* expression, and then in the remaining dermomyotomal lips (Buckingham and Rigby, 2014). Several transcriptional regulators that control the activation of MRF expression in the epaxial and hypaxial lips of the dermomyotome have been identified. In the dermomyotome, members of the *Sine oculis homeobox* (SIX) and *Eyes absent homologue* (EYA) protein families are co-expressed with PAX3 and regulate its expression (Grifone et al., 2005, 2007). Moreover, overexpression of SIX1 in chicken embryos actives *Pax3* expression (Heanue et al., 1999). Compound mouse mutants for *Six1;Six4* and *Eya1;Eya2* lose *Pax3* expression and show a similar phenotype to that of *Pax3*-deficient embryos. SIX and EYA proteins regulate myogenesis hypaxially via regulation of *Pax3* expression (Grifone et al., 2007) but also epaxially, where SIX1 and SIX4 directly bind to MRF4 regulatory regions controlling its expression (Grifone et al., 2005). The epaxial dermomyotome domain is also regulated by the transcription factor *Dmrt2*, whose expression is downregulated in *Pax3*-deficient embryos and is required for *Myf5* expression (Seo, 2007; Sato et al., 2010).

In the mouse, a naturally occurring mutation on the *Pax3* allele was identified as “*splotch*” and homozygous for this mutation are embryonic lethal (Auerbach, 1954). In these embryos, somatic boundaries are disturbed as observed with changes in Ephrin type-A receptor 4 (*Epha4*) expression (Schubert et al., 2001). Genetically modified mouse lines containing different *Pax3* alleles have further contributed to understanding the role of PAX3 in myogenesis and to the discovery of some of its direct target genes. The *Pax3* gain-of-function allele *Pax3-FKHR* that encodes a fusion protein, comprising the PAX3 DNA binding domain and the transcriptional activation domain of FOXO1, rescues the *Pax3*-mutant phenotype showing that PAX3 functions as a transcriptional activator (Relaix et al., 2003). Heterozygous mouse embryos expressing the *Pax3-ERD* allele, where the PAX3-Engrailed fusion protein acts as a transcriptional repressor, show an attenuated phenotype (compared to *Pax3*-null embryos) at the level of the hypaxial somite derivatives (Bajard et al., 2006; Relaix et al., 2006). This fusion protein that comprises the PAX3 DNA binding domain and the transcriptional repressor domain of the *Drosophila* engrailed gene behaves as a dominant-negative form of PAX3 in cultured cells (Relaix et al., 2006) and genetically as a hypomorphic allele (Bajard et al., 2006). The modulation of expression of genes operating downstream of PAX3 according to PAX3 transcriptional activity in the presence of each of these alleles, allowed the identification of PAX3 direct target genes like *Myf5*, *Fgfr4, Itm2a*, *Dmrt2*, and *c-Met* (Epstein et al., 1996; Bajard et al., 2006; Lagha et al., 2008, 2013; Sato et al., 2010). Moreover, several paraxial mesoderm markers such as *Meox1*, *Paraxis*, *c-Met* and *Dll1* were upregulated in murine embryonic stem cells (ESCs) upon PAX3 induction (Magli et al., 2013). Interestingly, P-Cadherin was identified as a PAX3-FKHR direct target gene in alveolar rhabdomyosarcoma samples, an aggressive childhood cancer of skeletal muscle (Thuault et al., 2013). In addition to its role as a transcription factor, PAX3 is involved in remodeling chromatin accessibility at myogenic loci (Boudjadi et al., 2018; Magli et al., 2019a). This occurs in cooperation with SIX4 and TEA domain family member 2 (TEAD2) for a well-defined myogenic commitment (Magli et al., 2019a).

The patterning of the myotome is a complex process that relies on cell rearrangements and tissue remodeling proteins including cadherins, fibronectin, collagens, tenascins, and laminins (Thorsteinsdóttir et al., 2011). Moreover, several structural proteins were upregulated in a transcriptomic analysis of the PAX3 gain-of-function allele *Pax3^*Pax*3–*FKHR/GFP*^* (Lagha et al., 2010) which led us to address the putative role of PAX3 upstream of the rearrangement of the myotome. The proper expression of the extracellular matrix-associated genes is required for the correct elongation and expansion of the myotome. Several cadherins are expressed in developing skeletal muscle: N-Cadherin (*CDH2*), R-Cadherin (*CDH4*), and M-Cadherin (*CDH15*) (Moore and Walsh, 1993; Rosenberg et al., 1997; Horikawa and Takeichi, 2001; Cinnamon et al., 2006). Cadherins are calcium-dependent cell-cell adhesion molecules that display preferentially homophylic interactions in neighboring cells. During development, N-Cadherin and R-Cadherin are expressed in several tissue types while M-cadherin is restricted to the skeletal muscle and cerebellum (Moore and Walsh, 1993; Rose et al., 1994; Rosenberg et al., 1997). Moreover, M-Cadherin expression becomes limited to satellite cells in adult muscles (Moore and Walsh, 1993; Irintchev et al., 1994), exhibiting a more specific expression pattern for skeletal muscle tissue compared to other cadherins. In addition, while N-Cadherin is expressed in the entire developing somite, M-Cadherin protein starts to be expressed at E10.5, specifically in the myotome, where it co-localizes with the structural protein Desmin (Moore and Walsh, 1993; Rose et al., 1994).

Here, we characterize the defective phenotype of the myotome in *Pax3*-mutant embryos and in embryos with gain- or loss-of-function alleles for *Pax3*. Moreover, we show that M-Cadherin expression is controlled by PAX3 transcriptional activity in the developing somite and further experiments lead us to propose that the gene for M-Cadherin is a target of PAX3.

## Materials and Methods

### Mouse Lines and Embryos

Mouse lines with the following alleles were used in this study: *Pax3^*nLacZ*^* (Relaix et al., 2003), *Pax3^*Pax*3–*ERD*–*IRESnLacZ*^* (referred to as *Pax3^*Pax*3–*ERD*^*) (Bajard et al., 2006), *Pax3^*Sp*^* (Relaix et al., 2003), *Pax3^*Pax*3–*FKHR*–*IRESnLacZ*^* (referred to as *Pax3^*Pax*3–*FKHR*^*) (Relaix et al., 2003), and *Pax3^*GFP*^* (Relaix et al., 2005). Embryos were staged according to Embryonic day (E) 0.5 as the day when the vaginal plug was observed. Embryos were fixed in 4% PFA at 4°C for 1 h and 30 min for immunostaining analysis and 15 min for X-GAL staining. Animals were handled according to the European Community guidelines, implementing the 3R rule. Protocols for the use of mouse embryos before the stage E14.5 are not subjected to further project validation, according to the guidelines of the ethic committee of the French Ministry.

### X-Gal Staining

X-GAL staining was performed as previously described (Relaix et al., 2003). Briefly, embryos were stained with an X-GAL-containing buffer for 4–16 h at 37°C with agitation. Embryos were then washed in PBS and post-fixed in 4% PFA overnight at 4°C.

### Immunostainings (Whole-Mount and Sections)

Whole-mount immunostainings were performed as previously described (Guris et al., 2001; Relaix et al., 2003) using the following antibodies: MF20 (1:300) that recognizes muscle myosin heavy chains, developed by D.A. Fischman and obtained from the Developmental Studies Hybridoma Bank developed under the auspices of the NICHD and maintained by The University of Iowa, Department of Biology Iowa City, IA 52242, and Desmin (Dako, 1:200). Immunostaining on sections were performed as previously described (Esteves de Lima et al., 2014, 2016). Embryos were included in gelatin/sucrose and snap-frozen. Sections of 12μm were fixed in PFA 4%, permeabilized with Triton 0.5% for 20 min and blocked for 1 h in 5% BSA IgG-free. Overnight incubation at 4°C was performed with the following antibodies: MF20 (1:300) developed by A. Kawakami was obtained from the Developmental Studies Hybridoma Bank developed under the auspices of the NICHD and maintained by the University of Iowa, Department of Biology Iowa City, IA 52242, MYOG (Santa Cruz, 1:50), cleaved CASP3 (Cell Signaling, 1:100), Desmin (Dako, 1:200), Laminin (Abcam, 1:1,000), β -GAL (Promega, 1:100) and M-Cadherin (BD Biosciences, 1:100). Alexa conjugated secondary antibodies (Thermo Fisher Scientific, 1:500) were incubated for 1 h at room temperature and nuclei were stained with DAPI (1:5,000) for 10 min.

### *In situ* Hybridization

Whole-mount *in situ* hybridization was performed as previously described (Tajbakhsh et al., 1997). The Cdh15 probe was obtained by PCR from embryonic tissues using the primers described in the Supplementary Table 1 and cloned into the pGEM-T easy vector (Promega). Probe preparation was performed by linearizing the vector with *Apa*I and synthetizing with SP6.

### Chromatin Immunoprecipitation and RT-qPCR Analysis

Wild-type embryos at E12.5 were collected, dissected for trunk and limb buds, removing head and internal organs. The ChIP protocol was performed as previously described (Harada et al., 2018) with the following modifications. Embryonic tissues were fixed with 0.5% formaldehyde in PBS 8 min at room temperature and mechanically disrupted with a 25G syringe. The fixation reaction was stopped with 1.25M Glycine. The tissue pellet was resuspended in ChIP buffer (Harada et al., 2018) and incubated for 15 min on ice. Chromatin digestion was performed with Micrococcal nuclease (Cell Signaling, #10011) for 40 min at 37°C. The supernatant containing 20 μg of DNA was incubated with 20 μL of magnetic beads (Thermo Fisher Scientific) and 5 μg of PAX3 antibody, developed by C.P. Ordahl and obtained from the Developmental Studies Hybridoma Bank developed under the auspices of the NICHD and maintained by The University of Iowa, Department of Biology Iowa City, IA 52242. After washing the beads, DNA was eluted and reverse cross-linked with 0.5 M NaCl for 4 h at 65°C and 2% proteinase K for 1 h at 50°C. DNA was purified using MicroChIP Diapure columns (Diagenode). Analyses were performed by RT-qPCR and the results expressed as a percentage of the input.

### Luciferase Enhancer Assay

To generate the pGL3-p34-TK-Luciferase (firefly luciferase) plasmid: the cassette (Relaix et al., 2003) containing the PAX3 responsive elements and the TK promoter (p34-TK) was excised with *Spe*I and *Hin*dIII from the p34-TK-nlacZ plasmid and cloned into the pGL3-TK-Luciferase plasmid (previously digested with *Nhe*I and *Hin*dIII to remove the TK promoter). To generate the pGL3-Peak-1-TK-Luciferase and the pGL3-Peak-2-TK-Luciferase plasmids Peak-1 and Peak-2 were amplified using primers containing *Sac*II restriction sites (Supplementary Table 1), sub-cloned into the pGEMTeasy vector, digested with *Sac*II and cloned into the pGL3-TK-Luciferase plasmid (previously digested with *Sac*II). For luciferase assays, pGL3 plasmids (TK-Luciferase (empty), p34-TK-Luciferase (positive control), Peak-1-TK-Luciferase or Peak-2-TK-Luciferase) vectors were co-transfected with the pCigPax3 expression vector (Relaix et al., 2003) or pCig (empty vector used for normalization) and pRL-CMV (renilla luciferase) for luminescence normalization into HEK 293T cells. Transfected cells were cultured for 48 h and subjected to luciferase assays using the Promega Dual-Luciferase Reporter Assay System.

## Results

### *Pax3*-Mutant Embryos Display Impaired Myotome Formation

**FIGURE 1:**
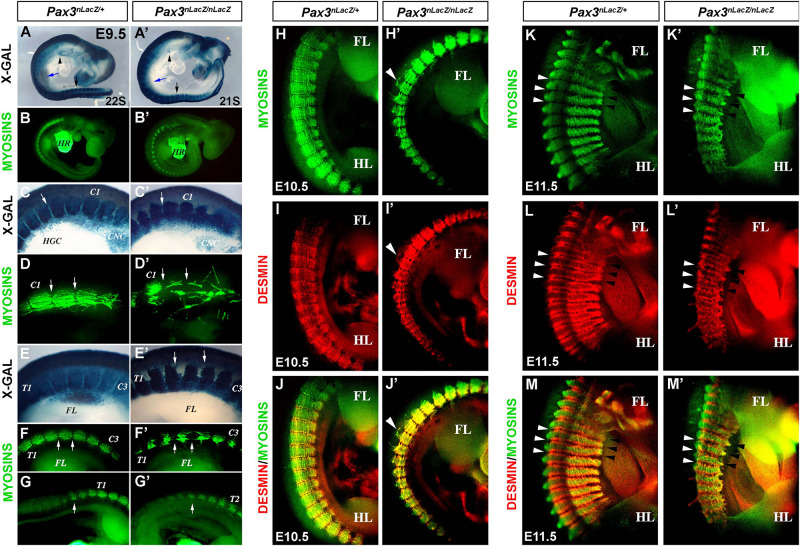
Characterization of the myotome of *Pax3*-mutant embryos. **(A,A′)** X-GAL staining of whole-mount control (*Pax3^*nLacZ/*+^*) and *Pax3*-mutant (*Pax3^*nLacZ/nLacZ*^*) embryos at E9.5 (arrow head, hypoglossal cord; blue arrow, forelimb; black arrow, hindlimb). **(B,B′)** Whole-mount immunostaining to visualize muscle Myosins in control (*Pax3^*nLacZ/*+^*) and *Pax3*-mutant (*Pax3^*nLacZ/nLacZ*^*) embryos at E9.5 (HR, heart). **(C,C′)** High magnification of X-GAL staining of whole-mount control (*Pax3^*nLacZ/*+^*) and *Pax3*-mutant (*Pax3^*nLacZ/nLacZ*^*) embryos in the cervical region at E9.5 (C1, Cervical Somite 1; HGC, hypoglossal cord; CNC, cardiac neural crest cells; arrow, somite boundary). **(D,D′)** High magnification of whole-mount immunostaining to visualize muscle Myosins in control (*Pax3^*nLacZ/*+^*) and *Pax3*-mutant (*Pax3^*nLacZ/nLacZ*^*) embryos at the cervical region at E9.5 (arrows, somite boundaries). **(E,E′)** High magnification of X-GAL staining of whole-mount control (*Pax3^*nLacZ/*+^*) and *Pax3*-mutant (*Pax3^*nLacZ/nLacZ*^*) embryos in the thoracic region at E9.5 (T1, Thoracic Somite 1; C3, Cervical Somite 3; arrows, Dorsal root ganglia lacking in the mutant embryo). **(F–G′)** High magnification of whole-mount immunostaining to visualize muscle Myosins in control (*Pax3^*nLacZ/*+^*) and *Pax3*-mutant (*Pax3^*nLacZ/nLacZ*^*) embryos in the thoracic region at E9.5 (T1, Thoracic Somite 1; T2, Thoracic Somite 2; C3, Cervical Somite 3; FL, forelimb; arrows, somite boundaries). **(H–M′)** Whole-mount immunostaining to visualize muscle Myosins **(H,H′,K,K′)**, Desmin **(I,I′,L,L′)** and the merge **(J,J′,M,M′)** in control (*Pax3^*nLacZ/*+^*) and *Pax3*-mutant (*Pax3^*nLacZ/nLacZ*^*) embryos at E10.5 **(H–J′)** and E11.5 **(K–M′)** (FL, forelimb; HL, hindlimb; white arrow heads, epaxial somite boundary; black arrow heads, hypaxial somite boundary).

The trunk muscles of *Pax3*-mutant embryos are severely affected (Bober et al., 1994; Goulding et al., 1994; Relaix et al., 2003). We aimed to dissect the early somite defects of myotome organization, which result in impaired trunk muscle formation. We analyzed the myotome of control (*Pax3^*nLacZ/*+^*) and *Pax3*-mutant (*Pax3^*nLacZ/nLacZ*^*) embryos, which contain the gene for β-galactosidase (*LacZ*) under the control of *Pax3* regulatory regions, allowing *Pax3* genetic lineage tracing by X-GAL staining. At E9.5, *Pax3*-null embryos lack colonization of the limb buds (blue and black arrows) and of the hypoglossal cord (arrowhead) by X-GAL + (*Pax3*-expressing) cells (Figures 1A–B′), as previously described (Relaix et al., 2003). We used the MF20 antibody that recognizes muscle Myosin heavy chains, to further characterize the structure and organization of myotomal muscle which at this stage has not yet formed multinucleated muscle fibers but is composed of differentiated myocytes, with organized muscle sarcomeres referred to here as fibers. While in control embryos the fibers of each myotome align and attach at the edges, in the *Pax3*-null embryos the fibers are detached and misaligned in both cervical and thoracic regions (Figures 1C–G′). This phenotype was more pronounced in the most anterior somites but defects could be observed all along the embryonic axis. The structural protein Desmin, which is expressed in both myoblasts and differentiated muscle cells, shows a regionalized pattern at the edges of the fibers in control embryos at E10.5, where it co-localizes with Myosins (Figures 1H–J). Strikingly, this regionalization is lost in *Pax3-*mutant embryos where it co-localizes with muscle Myosins all over the myotome (Figures 1H′–J′); indicating that patterning of the myotome fibers is affected. Moreover, we observed that in the absence of PAX3 there is an overlap between adjacent somites as observed with Desmin immunostaining (Figures 1K–M′, arrows) and with X-GAL staining (Supplementary Figures 1A–B′). At early stages of somitogenesis, PAX3 also acts as a survival factor for hypaxial dermomyotome which is reduced in *Pax3*-mutant embryos (Borycki et al., 1999; Relaix et al., 2004). To analyze if the reduced Myosin staining observed in mutant embryos was due to loss of myocytes and/or reduced MYOG + cell number at a later time-point (E12.5) we quantified the number of MYOG + cells in the myotomes of *Pax3*-deficient embryos and controls. We observed that MYOG + cell numbers (normalized on the unit area) are not changed in the myotomes in the absence of PAX3 (Supplementary Figures 1C–E). Moreover, MYOG + cells are not undergoing apoptosis as analyzed by cleaved Caspase 3 (CASP3) immunostaining (Supplementary Figures 1C″,D″). Taken together, we conclude that loss of *Pax3* leads to defective myotome formation associated with mis-localization of structural proteins.

### Impaired Myotome Formation in *Pax3*-Mutant Embryos Is Associated With Defective Basal Lamina Organization

**FIGURE 2:**
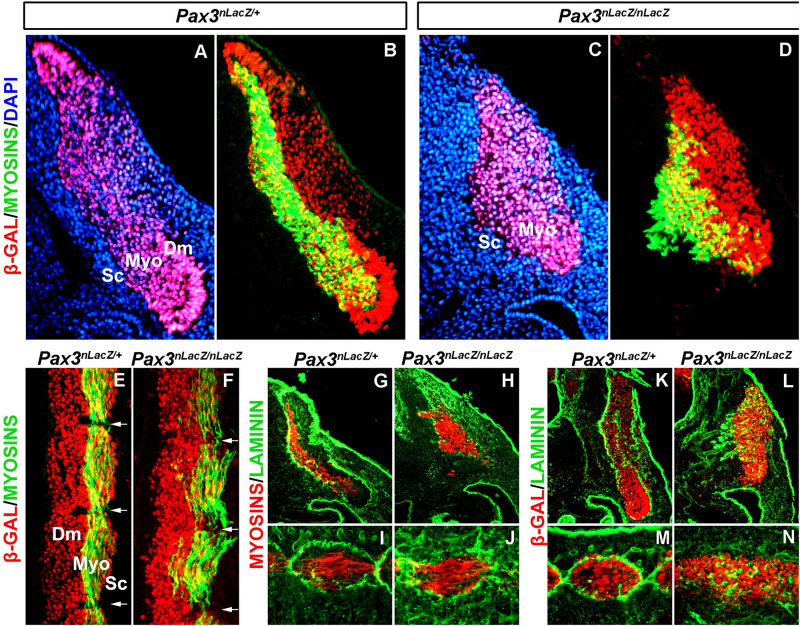
Characterization of the basal lamina in the myotome of *Pax3*-mutant embryos. **(A–D)** Immunostaining on transverse sections at the anterior thoracic level of E11.5 embryos to visualize myosins (green), β-GAL (red) that labels the PAX3 genetic lineage and DAPI to visualize the nuclei (blue) in control (*Pax3^*nLacZ/*+^*) **(A,B)** and *Pax3*-mutant (*Pax3^*nLacZ/nLacZ*^*) **(C,D)** embryos (Dm, dermomyotome; Myo, myotome; Sc, sclerotome). **(E,F)** Immunostaining on longitudinal sections (thoracic somites) of E11.5 embryos to visualize muscle Myosins (green) and β-GAL (red) that labels cells that have or would have expressed *Pax3* in control (*Pax3^*nLacZ/*+^*) **(E)** and *Pax3*-mutant (*Pax3^*nLacZ/nLacZ*^*) **(F)** embryos (Dm, dermomyotome; Myo, myotome; Sc, sclerotome). **(G,J)** Immunostaining on transverse **(G,H)** and longitudinal **(I,J)** sections of E11.5 embryos to visualize muscle Myosins (red) and Laminin (green) in control (*Pax3^*nLacZ/*+^*) **(G,I)** and *Pax3*-mutant (*Pax3^*nLacZ/nLacZ*^*) **(H,J)** embryos. **(K,N)** Immunostaining on transverse **(K,L)** and longitudinal **(M,N)** sections (thoracic somites) of E11.5 embryos to visualize β-GAL that labels the PAX3 genetic lineage (red) and Laminin (green) in control (*Pax3^*nLacZ/*+^*) **(K,M)** and *Pax3*-mutant (*Pax3^*nLacZ/nLacZ*^*) **(L,N)** embryos.

To further characterize myotome patterning defects, we analyzed sections of E11.5 embryos. In control *Pax3^*nLacZ/*+^* embryos, PAX3-derived cells (β-GAL immunostaining) are present in the epaxial and hypaxial dermomyotome, which display an epithelialized structure, and at the level of the myotome, resulting from the migration of the dermomyotome progenitor cells. Of note, the high stability of the β-GAL protein allows the fate-tracing of PAX3-expressing cells (Figures 2A,B; Relaix et al., 2005). At the level of the myotome, traced β-GAL + cells express muscle Myosins, showing the differentiation of myoblasts, and also, by this stage, the formation of multinucleated muscle fibers as a result of fusion (Figures 2A,B). In *Pax3^*nLacZ/nLacZ*^* embryos, the epithelialized structure of the dermomyotome is lost and myotome formation is severely impaired (Figures 2C,D). In addition, longitudinal sections of the somites confirm the mis-alignment and detachment of the myotome fibers to the edges (Figures 2E,F). Because basal lamina proteins play important structural roles, we analyzed Laminin localization in control and *Pax3-mutant* embryos. While in control somites Laminin is surrounding the dermomyotome and the myotome, delimiting the myotome fibers (Figures 2G,I,K,M), *Pax3*-null embryo somites lack this ordered expression of Laminin, with the fibers expanding beyond individual myotome domains (Figures 2H,J,L,N). These results show that myotome organization is tightly associated with basal lamina deposition and suggest that Laminin localization is associated with myotome fiber orientation and boundary definition.

### *Pax3^*Pax*3–*ERD/Sp*^* Mutant Embryos Present Severely Reduced Myotomes

**FIGURE 3:**
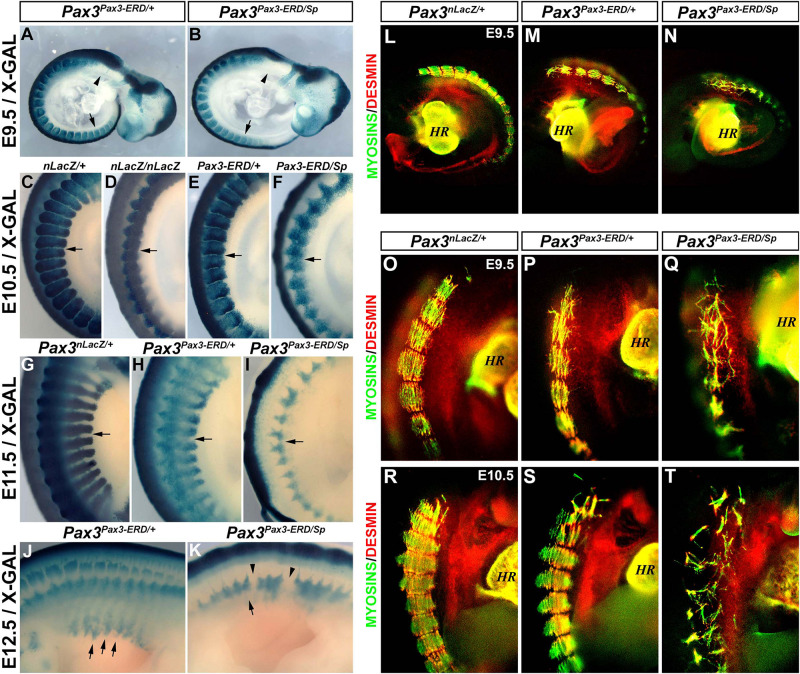
Characterization of the myotome of *Pax3^*Pax*3– *ERD/*+^* and *Pax3^*Pax*3– *ERD/Sp*^* embryos. **(A,K)** X-GAL staining of whole-mount control (*Pax3^*nLacZ/*+^*) **(C,G)**, *Pax3*-mutant (*Pax3^*nLacZ/nLacZ*^*) **(D)**, *Pax3^*Pax*3– *ERD/*+^* **(A,E,H,J)**, and *Pax3^*Pax*3– *ERD/Sp*^* **(B,F,I,K)** embryos at E9.5 **(A,B)**, E10.5 **(C–F)**, E11.5 **(G–I)**, and E12.5 **(J,K)**. **(L–T)** Whole-mount immunostaining to visualize muscle Myosins (green) and Desmin (red) in control (*Pax3^*nLacZ/*+^*) **(L,O,R)**, *Pax3^*Pax*3– *ERD/*+^* **(M,P,S)** and *Pax3^*Pax*3– *ERD/Sp*^* **(N,Q,T)** embryos at E9.5 **(L–Q)** and E10.5 **(R–T)** (HR, heart).

Heterozygous mouse embryos expressing the *Pax3^*Pax*3–*ERD/*+^* allele display a hypomorphic phenotype and this allele functions as a dominant-negative form of PAX3 (Bajard et al., 2006; Relaix et al., 2006). In order to analyze if the presence of a dominant-negative allele combined with a null allele for PAX3 (*splotch*) displayed a more severe phenotype than that of *Pax3*-null embryos we analyzed the somite phenotype of *Pax3^*Pax*3–*ERD/*+^* and *Pax3^*Pax*3–*ERD/Sp*^* embryos from E9.5 to E12.5. As previously observed, *Pax3^*Pax*3–*ERD/*+^* embryos stained with X-GAL show a few cells migrating at the level of the hypoglossal cord (arrow head) and hindlimb (arrow) at E9.5 (Figure 3A; Bajard et al., 2006). This hypomorphic phenotype is distinct from the one observed in *Pax3^*nLacZ/nLacZ*^* (Figure 1A) and *Pax3^*Pax*3–*ERD/Sp*^* embryos (Figure 3B) which do not present any migratory progenitor cells. At E10.5, we observed that the somite boundaries are partially maintained in the *Pax3^*Pax*3–*ERD/*+^* embryos but lost in *Pax3^*nLacZ/nLacZ*^* and in *Pax3^*Pax*3–*ERD/Sp*^* embryos, with a more severe phenotype in the latter (Figures 3C–F). The loss of somite boundaries and impaired development of the myotomes in *Pax3^*Pax*3–*ERD/Sp*^* embryos is very severe at E11.5 and 12.5, while the defects are milder in *Pax3^*Pax*3–*ERD/*+^* embryos (Figures 3G–K). To further analyze the phenotype of the myotome fibers in these embryos, we performed whole-mount fluorescent immunostainings for Desmin and muscle Myosins. While *Pax3^*Pax*3–*ERD/*+^* embryos present a similar phenotype to that of *Pax3^*nLacZ/nLacZ*^* embryos (Figures 1H′–J′) with disorganized myotome fibers and Desmin localization not restricted to the edges of the fibers (Figures 3L,M,O,P,R,S), *Pax3^*Pax*3–*ERD/Sp*^* mutant embryos display a severe loss of myotome fibers at E9.5 and E10.5 (Figures 3N,Q,T). Of note, the dominant-negative function of the *Pax3^*Pax*3–*ERD*^* allele may also affect the expression of PAX7 targets genes, which can contribute to the severity of the phenotype observed. We conclude that combining the allele that encodes *Pax3-engrailed* with a null *Pax3* allele leads to a more severe phenotype than that of *Pax3*-null alone, consistent with the hypothesis that PAX3-Engrailed behaves as a dominant-negative form of PAX3.

### PAX3 Transcriptional Activity Modulates M-Cadherin Expression

**FIGURE 4:**
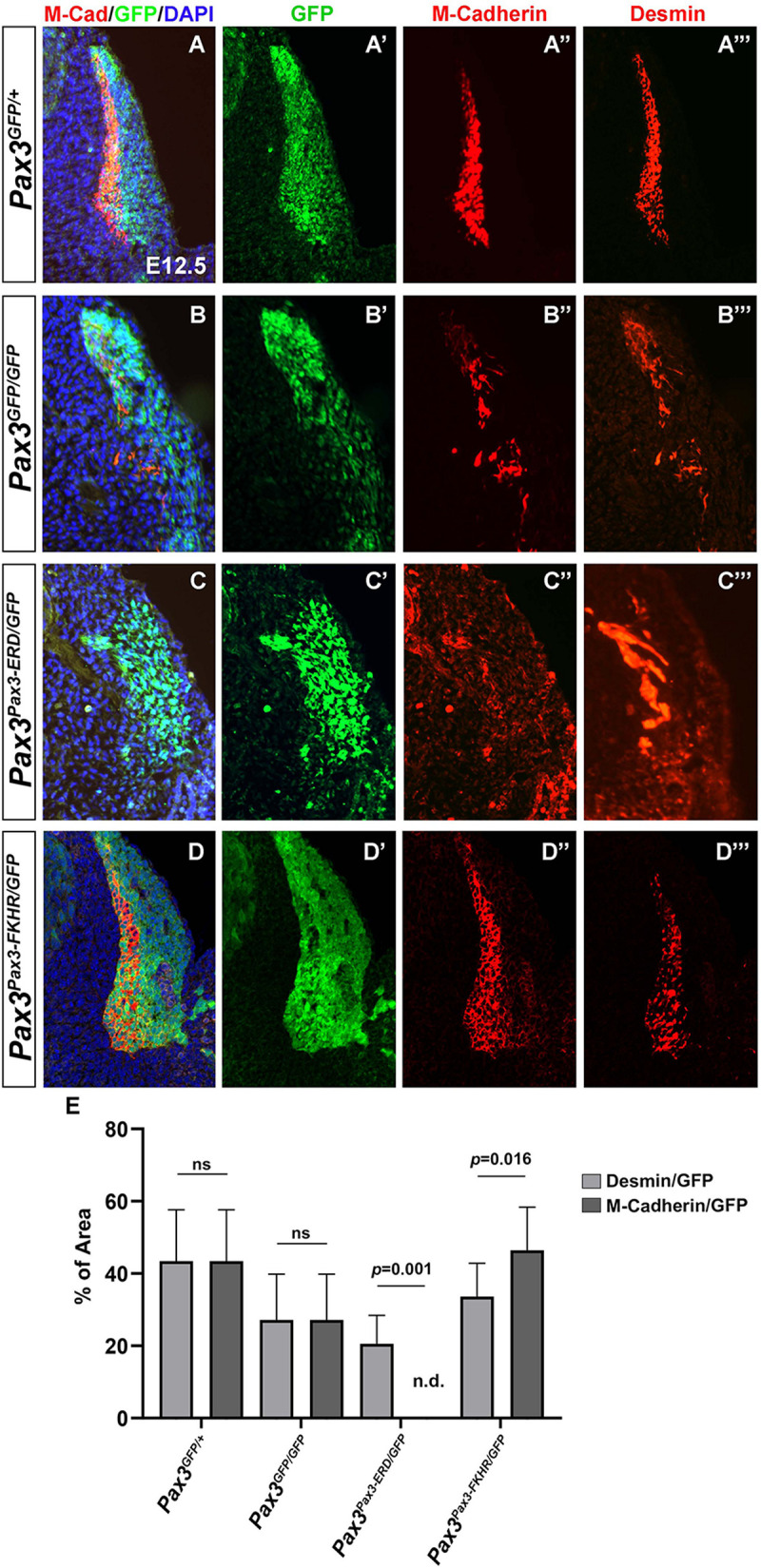
Characterization of M-Cadherin expression in the myotome of embryos with different *Pax3* alleles. **(A–D)** Immunostaining on transverse sections of E12.5 embryos to visualize M-Cadherin (red), GFP (green) that labels the PAX3 genetic lineage, Desmin (far-red and represented in red in the panels **A″′,B″′,C″′,D″′**) and DAPI to visualize the nuclei (blue) in control (*Pax3^*GFP*/+^*) **(A)**, *Pax3*-mutant (*Pax3^*GFP/GFP*^*) **(B)**, *Pax3^*Pax*3– *ERD/GFP*^* **(C)**, and *Pax3^*Pax*3– *FKHR/GFP*^* **(D)** embryos. **(A′,B′,C′,D′)** Immunostaining to visualize GFP (green) that labels the PAX3 lineage (same section as in **A–D**), in control (*Pax3^*GFP/*+^*) **(A′)**, *Pax3*-mutant (*Pax3^*GFP/GFP*^*) **(B′)**, *Pax3^*Pax*3– *ERD/GFP*^* **(C′)**, and *Pax3^*Pax*3– *FKHR/GFP*^* **(D′)** embryos. **(A″,B″,C″,D″)** Immunostaining to visualize M-Cadherin (red) (same section as in **A–D**) in control (*Pax3^*GFP/*+^*) **(A″)**, *Pax3*-mutant (*Pax3^*GFP/GFP*^*) **(B″)**, *Pax3^*Pax*3– *ERD/GFP*^* **(C″)**, and *Pax3^*Pax*3– *FKHR/GFP*^* **(D″)** embryos. **(A″′,B″′,C″′,D″′)** Immunostaining to visualize Desmin (red) (same section as in **A–D**) in control (*Pax3^*GFP/*+^*) **(A″′)**, *Pax3*-mutant (*Pax3^*GFP/GFP*^*) **(B″′)**, *Pax3^*Pax*3– *ERD/GFP*^* **(C″′)**, and *Pax3^*Pax*3– *FKHR/GFP*^* **(D″′)** embryos. **(E)** Quantification of the percentage of the surface area of M-Cadherin vs. GFP and Desmin vs. GFP in the different *Pax3* alleles. Graph represents the mean with standard deviations. The *p*-value was calculated with a two-tailed paired *t*-test.

In order to identify putative targets of PAX3 that could be involved in myotome alignment, we revisited the transcriptomic analysis performed on embryonic PAX3 + cells (*Pax3^*GFP/*+^*) and cells presenting a PAX3 gain-of-function allele (*Pax3^*Pax*3–*FKHR/GFP*^*) (Supplementary Figure 2A; Lagha et al., 2010). We screened for adhesion molecules upregulated in somites of *Pax3^*Pax*3–*FKHR/GFP*^* compared to *Pax3^*GFP/*+^* embryos and identified M-Cadherin. We analyzed the profile of M-Cadherin protein expression in control and *Pax3*-mutant (*Pax3^*GFP/GFP*^*) myotomes in E12.5 embryos and confirmed that M-cadherin protein levels are decreased in *Pax3*-deficient embryos (Figures 4A,B). However, since *Pax3*-mutants display a severe myotome phenotype, we wished to determine whether decreased M-cadherin protein was associated with the reduced myotome in *Pax3*-deficient embryos or directly linked to the loss of PAX3 protein expression. We therefore compared M-Cadherin levels to those of Desmin (that was not found to be a target of PAX3 in the published datasets), that are co-expressed in the myotome (Figures 4A–A″′). To do so, we quantified the surface area of M-Cadherin and Desmin and normalized to that of the GFP, which represents the PAX3 genetic lineage, in the myotome and the dermomyotome. In *Pax3*-mutant embryos, decreased M-Cadherin protein levels are comparable to those of Desmin (Figures 4B–B″′,E). Next, we used the various *Pax3* alleles to evaluate whether M-Cadherin expression was modulated by PAX3 transcriptional activity *in vivo*. We examined embryos with PAX3 gain- and loss-of-function alleles (*Pax3^*Pax*3–*FKHR/GFP*^*, *Pax3^*Pax*3–*ERD/GFP*^*, respectively) (Relaix et al., 2003; Bajard et al., 2006). We observed that in *Pax3^*Pax*3–*ERD/GFP*^* embryos, M-Cadherin expression is abrogated, although Desmin is still expressed (Figures 4C–C″′,E). Conversely, in *Pax3^*Pax*3–*FKHR/GFP*^* embryos, M-Cadherin surface area is increased compared with that of Desmin (Figures 4D–D″′,E), indicating that PAX3 can drive M-Cadherin expression. Because M-Cadherin protein synthesis occurs with a delay of 2–3 days to that of mRNA production (Rose et al., 1994) we performed *in situ hybridization* in control (*Pax3^*GFP/*+^*) and *Pax3*-deficient embryos (*Pax3^*GFP/GFP*^*) at E10.5 to investigate if changes in protein levels were associated with early transcriptional variations. We confirmed that E10.5 *Pax3*-mutant embryos display a reduced expression of M-Cadherin (*Cdh15*) compared to control embryos (Supplementary Figures 2B–C′). Taken together these data indicate that PAX3 transcriptional activity regulates M-Cadherin expression in the myotome.

### PAX3 Directly Binds to M-Cadherin Putative Regulatory Regions

**FIGURE 5:**
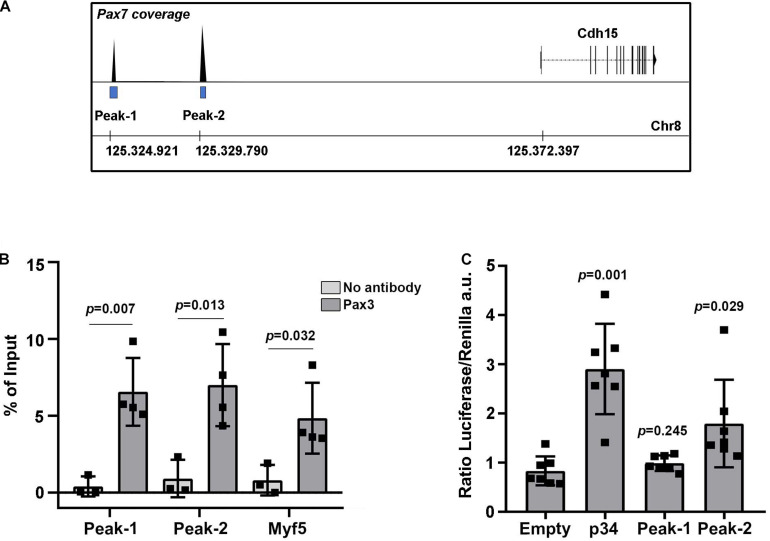
Identification of M-Cadherin as a direct target of PAX3. **(A)** Schematic representation of the detected peaks bound by PAX7 from Soleimani et al. (2012) upstream of the M-Cadherin gene (*Cdh15*) locus. **(B)** ChIP-RT-qPCR for PAX3 on Peak-1 and Peak-2 and on the positive control at −57 kb upstream of *Myf5*. Graph represents the mean with standard deviations. The *p*-value was calculated with a two-tailed paired *t*-test. **(C)** Quantification in arbitrary units of the ratio between luciferase and renilla (transfection control) in the presence of an expression vector for PAX3 and a plasmid containing the luciferase gene under the control of the p34 sequence, as a positive control, the Peak-1 sequence, the Peak-2 sequence or no potential PAX3 binding sequence (empty). Graph represents the mean with standard deviations. The *p*-value was calculated with a two-tailed paired *t*-test.

Changes in PAX3 transcriptional activity modulate the levels of M-Cadherin protein in the myotome. To address whether PAX3 directly regulates the M-Cadherin gene (*Cdh15*) expression, we scanned for PAX3 and PAX7 genomic binding sites in previously published genome-wide binding analyses performed in adult myoblasts (Soleimani et al., 2012). We identified two significant peaks for PAX7 binding at −47.5 Kb (Peak-1) and −42.6 Kb (Peak-2) upstream of *Cdh15* (Figure 5A), but no significant peaks for PAX3. Previous studies have shown that PAX3 and PAX7 display divergent functions in postnatal myogenesis (Relaix et al., 2006; Soleimani et al., 2012) while they display highly overlapping functions during development (Relaix et al., 2004). We therefore tested if these genomic regions were bound by PAX3 during development. We performed chromatin immunoprecipitation (ChIP) to validate PAX3 binding to these regions using wild type E12.5 embryonic tissues (limb buds and trunk). We analyzed the ChIP results by RT-qPCR and used the previously identified PAX3 binding site, −57 kb upstream of the *Myf5* myogenic factor gene, as a positive control (Bajard et al., 2006). We observed a significant enrichment of PAX3 bound to Peak-1 and Peak-2 as well as to the *Myf5* site, compared to the negative control (no antibody) (Figure 5B). Then, to examine whether these putative regulatory regions display an enhancer activity, we performed luciferase assays in non-myogenic cells. We cloned the sequences of Peak-1 and Peak-2 into a plasmid containing a minimal promoter regulating luciferase gene expression and used the same plasmid containing the p34 sequence (that contains multimers of the consensus PAX3 binding site) (Relaix et al., 2004) as a positive control. We performed these enhancer activity assays in a non-myogenic cell line (HEK 293T) to determine the transactivation capacity of PAX3 in the absence of myogenic co-factors. In the presence of a PAX3 expression vector, in HEK 293T cells we observed that Peak-2 significantly increases the levels of luciferase expression compared to a control plasmid containing only the minimal promoter (empty) (Figure 5C). By contrast, Peak-1, did not show any significant changes in driving luciferase expression compared to the control (Figure 5C). We conclude that PAX3 significantly binds Peak-1 and Peak-2, but only the binding to Peak-2 can activate and regulate transcription. Altogether, these data suggest that the M-Cadherin gene is a target of PAX3 during early myogenic development.

## Discussion

Embryos lacking the myogenic master regulator PAX3 display severe trunk muscle defects, which are disorganized or absent, and a complete loss of muscles of migratory origin (limb, tongue, diaphragm) (Bober et al., 1994; Goulding et al., 1994; Relaix et al., 2003). We observed that the formation of the myotome, which is the primitive muscle required for subsequent axial muscle patterning, is strongly affected in *Pax3*-deficient embryos, displaying disorganized myotome fibers compared to controls. In addition, in these embryos, the basal lamina surrounding the dermomyotome and the myotome loses the organized pattern observed in control embryos. It was previously shown that PAX3 modulates the expression of several laminins and integrins, some of which lie genetically downstream of the PAX3 direct target genes *Dmrt2* and *Myf5* (Lagha et al., 2010). These proteins play a major role in dermomyotome patterning and contribute to the formation of the basal lamina that delimits the myotome (Bajanca et al., 2006). These studies indicate that PAX3 regulates the organization of the myotome by modulating the expression of structural proteins. Our work further supports this hypothesis, showing that blocking PAX3 transcriptional activity leads to a severe myotome phenotype associated with changes in the cell adhesion molecule M-Cadherin. In addition to its transcription factor role, PAX3 was recently shown to regulate myogenesis by remodeling chromatin accessibility in loci that contain PAX3 binding sites (Magli et al., 2019a). Moreover, in the presence of the transcription factor LDB1, the *Pax3* locus forms specific topologically associated domains, which allows myogenic activity (Magli et al., 2019b). One cannot exclude that PAX3 might be modulating M-Cadherin expression via chromatin architectural modifications.

The proper development of the myotome requires the orchestrated patterning of the extracellular matrix and the regionalized localization of the transmembrane proteins that mediate cell-cell contact. *Pax3*-mutant embryos display a suppression of the expansion of the myotome. This phenotype is similar to the one observed when a N-Cadherin truncated protein, lacking the intracellular domain, is ectopically expressed in chick developing somites (Horikawa and Takeichi, 2001). In fact, since this molecule acts as a dominant-negative form, the activity of other cadherins might also be affected in these conditions. Interestingly, the lack of the extracellular domain of N-Cadherin in the same experimental design does not affect myotome expansion, which suggests that the intracellular downstream pathway of cadherins seems to be crucial for autonomous myotome cell rearrangement (Horikawa and Takeichi, 2001). Although, the intersomitic boundaries remain disturbed, showing that the extracellular domain of cadherins is required for somite boundary definition, within the myotome, the intracellular domain of cadherins is sufficient for its expansion (Horikawa and Takeichi, 2001).

We identified M-Cadherin as a protein regulated by PAX3 transcriptional activity. The levels of M-Cadherin in the myotome change according to the different *Pax3* alleles analyzed. In addition, PAX3 directly binds to putative regulatory regions of the M-Cadherin gene locus. Mutant embryos lacking M-Cadherin do not display a myotome or other skeletal muscle phenotype (Hollnagel et al., 2002). In addition, the development of the cerebellum, where M-Cadherin is also expressed, is not affected but it has increased expression levels of N-Cadherin, suggesting that a compensatory mechanism by other cadherins can take place in the absence of M-Cadherin (Hollnagel et al., 2002). As expected, muscle-specific M-Cadherin mutant embryos do not show skeletal muscle defects (Goel et al., 2017). Furthermore muscle-specific N-Cadherin mutants or the double conditional mutant for M-Cadherin and N-Cadherin do not show developmental defects (Goel et al., 2017). However, other cadherins, like R-Cadherin are also expressed in the developing myotome and could explain this phenotype through a compensatory mechanism.

Our study shows that M-Cadherin is expressed in the myotome and that its expression is modulated by PAX3, and while not essential for myotome development, our data suggests it contributes to myotome proper formation.

## Data Availability Statement

All datasets generated for this study are included in the article/Supplementary Material, further inquiries can be directed to the corresponding author/s.

## Ethics Statement

Animals were handled according to the European Community guidelines, implementing the 3R rule. Protocols for the use of mouse embryos before the stage E14.5 are not subjected to further project validation, according to the guidelines of the Ethic Committee of the French Ministry.

## Author Contributions

JEdL and FR designed, performed, and analyzed the experiments and wrote the manuscript. RBA, MM, and DR performed the experiments. MB and FR supervised the experiments and oversaw the project. MB edited and commented on the manuscript.

## Conflict of Interest

The authors declare that the research was conducted in the absence of any commercial or financial relationships that could be construed as a potential conflict of interest.
