# Supplementary material for: M-Cadherin Is a PAX3 Target During Myotome Patterning
Source: Front Cell Dev Biol. 2021 Apr 1;9:652652. doi: 10.3389/fcell.2021.652652 (PMC8047199; doi:10.3389/fcell.2021.652652)
Supplement: Supplementary file 1 [file Data_Sheet_1.docx]

**Supplementary Material**

**Esteves de Lima *et al.*, *M-Cadherin* is a PAX3 target during myotome patterning**

**
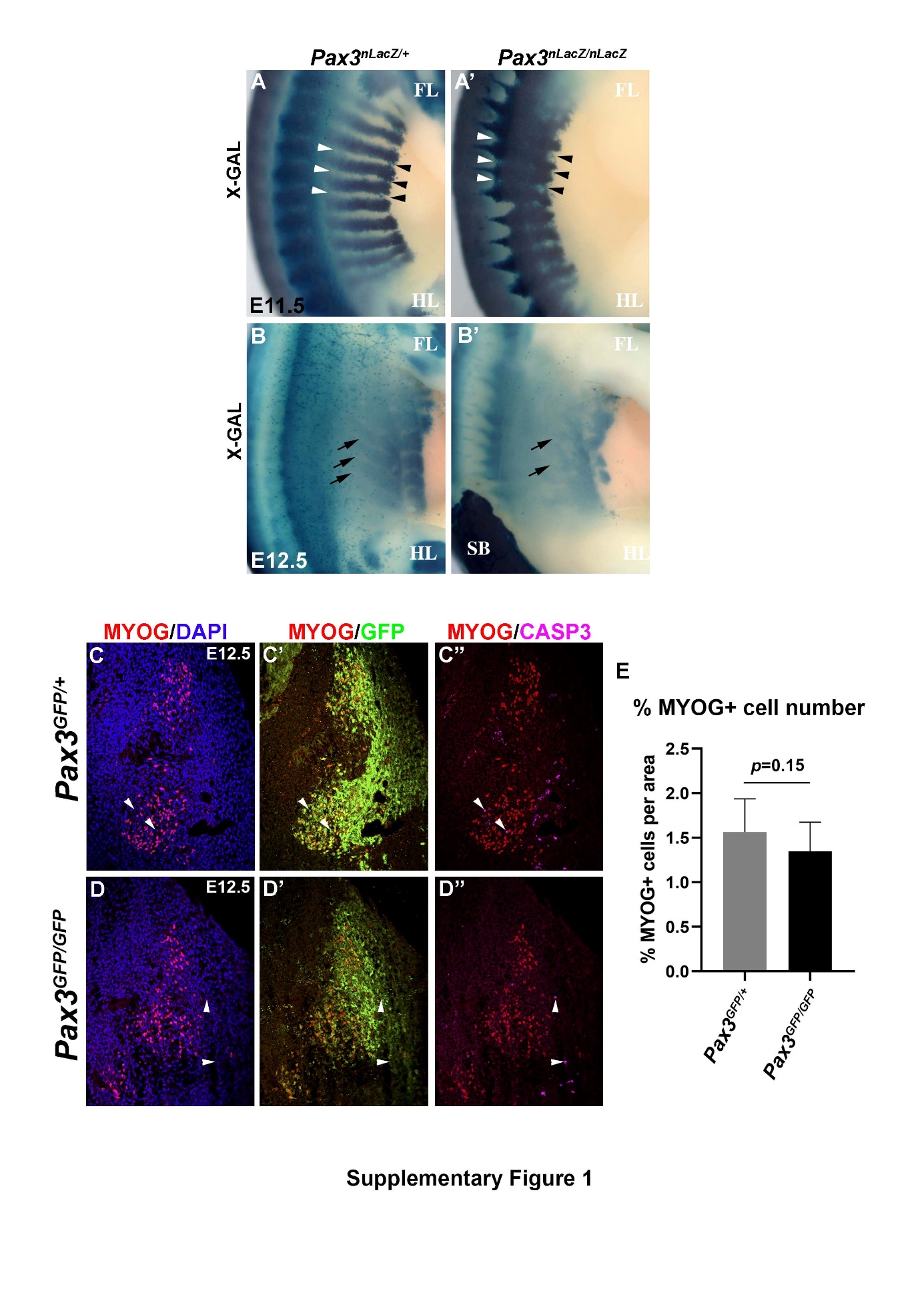
**

**Supplementary Figure 1 – X-Gal staining and MYOG+ cell quantification of *Pax3*-mutant embryos.** (A-B’) High magnification of X-GAL staining of whole-mount control (*Pax3^nLacZ/+^*) and *Pax3*-mutant (*Pax3^nLacZ/nLacZ^*) embryos at E11.5 (A, A’) and at E12.5 (B, B’) (FL, forelimb; HL, hindlimb; black arrow heads, hypaxial somite boundary; white arrow heads, epaxial somite boundary; SB, *spina bifida*; arrows). (C-D’’) Immunostaining to visualize MYOG (red), GFP (green) and CASP3 (purple) in control (*Pax3^GFP/+^*) (C,C’,C’’) and *Pax3*-mutant (*Pax3^GFP/GFP^*) (D,D’,D’’) embryos (arrowheads, MYOG-CASP3+ cells). (E) Quantification of the of the MYOG+ cell number normalized on the unit area in control and *Pax3*-deficient embryos. Graph represents the mean with standard deviations. The *p*-value was calculated with a two-tailed paired t-test.


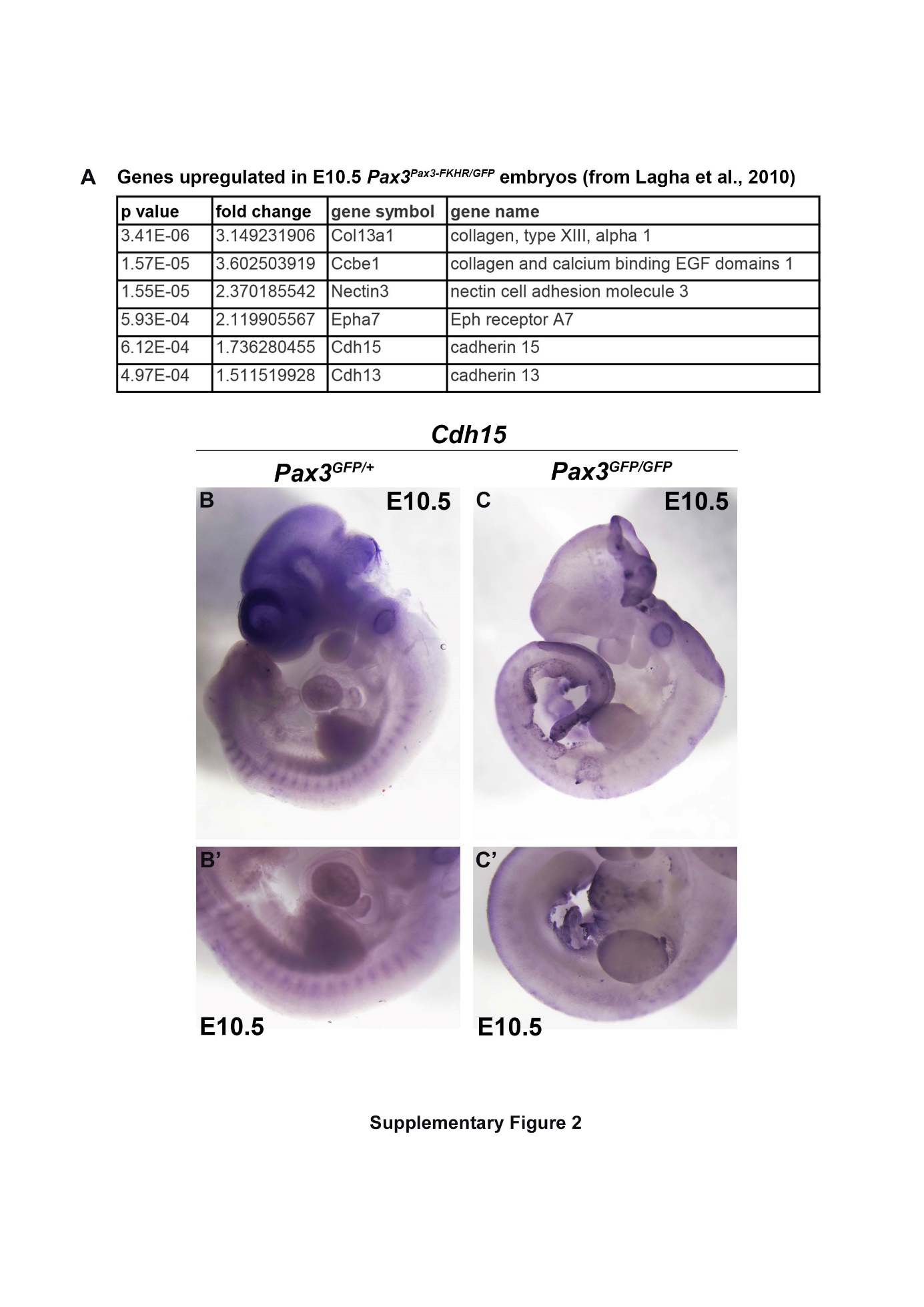


**Supplementary Figure 2 – M-Cadherin (*Cdh15*) expression in *Pax3*-mutant embryos.** (A) List of genes associated with cell interaction and upregulated in E10.5 embryos in *Pax3^Pax3-FKHR/GFP^* compared to *Pax3^GFP/+^* (from Lagha et al., 2010). (B-C’) Whole-mount in situ hybridization for M-Cadherin (*Cdh15*) in *Pax3^GFP/+^* (B, B’) and in *Pax3^GFP/GFP^* (C, C’) embryos at E10.5.

| ***In situ hybridization* primers** | | |
| --- | --- | --- |
|  | Forward Primer (5’-3’) | Reverse Primer (5’-3’) |
| Cdh15 | TGTGTCTGAGAACCACAAACGC | ACGTCCACTCCACTGACA |
| **ChIP-RT-qPCR primers** | | |
|  | Forward Primer (5’-3’) | Reverse Primer (5’-3’) |
| Peak-1 | CCGCGGATGCTTGCTTAGGGTTAGGAC | CCGCGGCAGGTGACAGTCACAG |
| Peak-2 | CCGCGGAACCACATGCTTGCATTACAG | CCGCGGTTGAAAAAAAAAAAAAAGAAAG |

**Supplementary Table 1 –** List of primers used in this study
